# Design, synthesis, docking, Hirshfeld surface analysis and DFT calculations of 2-methylxanthen-9-with the FtsZ protein from Staphylococcus aureus

**DOI:** 10.6026/97320630017393

**Published:** 2021-03-31

**Authors:** V Lakshmi Ranganatha, Mallikarjunaswamy C, Jagadeep Chandra S, Ramith Ramu, Prithvi S Shirahatti, Naveen Kumar, Sowmya BP, Hussien Ahmed Khamees, Mahendra Madegowda, Shaukath Ara Khanum

**Affiliations:** 1 Department of Chemistry, The National Institute of Engineering, Manandavadi Road, Mysuru 570008, Karnataka, India; 2 PG Department of Chemistry, JSS College of Arts, Commerce and Science, Ooty Road, Mysuru - 570025, Karnataka, India; 3 Department of Microbiology, School of Life Sciences, JSS Academy of Higher Education and Research, SS Nagar, Mysuru - 570015, karnataka, India; 4 Department of Biotechnology and Bioinformatics, School of Life Sciences, JSS Academy of Higher Education and Research, SS Nagar, Mysuru - 570015, Karnataka, India; 5 Department of Biotechnology, Teresian College, Siddhartha Nagara, Mysuru-570011, karnataka, India; 6 Department of Chemistry, Sri Dharmasthala Manjunatheshwara College, Ujire - 574240, karnataka, India; 7 Department of Studies in Physics, Manasagangotri, University of Mysore, Mysuru-570006, Karnataka, India; 8 Department of Chemistry, Yuvaraja's College (Autonomous), University of Mysore, Mysuru - 570 005, karnataka, India

**Keywords:** Synthesis, 2-Methylxanthen-9-one, antimicrobial, docking, DFT

## Abstract

It is of interest to document the design, synthesis, docking, Hirshfeld surface analysis and DFT calculations of 2-methylxanthen-9-with the FtsZ protein (PDB ID: 3VOB) from Staphylococcus aureus for antimicrobial applications. We report the quantitative structure function data in this context.

## Background

Xanthones are natural or synthetic compounds which are structurally related to anthraquinones and among these mitoxantrone is a well-established anti-cancer drug. [1, 2] Extracts of the pericarp of ripe fruits possess immuno modulating [3], anti-bacterial [4], anti-mutagenic [5,6], anti-cancer [7] and other pharmacological activities. It is known that naturally occurring pyranoxanthones are more active than dihydropyrano xanthones in terms of their biological activities [8]. Xanthones also concern semi-synthetic and synthetic heterocyclic compounds with the dibenzo-γ-pyrone scaffold. Interest on xanthone analogues has been growing considerably due to the wide range of pharmacological applications exhibited by this group of compounds, including anticancer, immuno modulation and other promising activities [9- 13]. Therefore, it is of interest to document the design, synthesis, docking, Hirshfeld surface analysis and DFT calculations of 2-methylxanthen-9-with the FtsZ protein (PDB ID: 3VOB) from Staphylococcus aureus for antimicrobial applications.

## Materials and Methods:

All solvents and reagents were purchased from Sigma Aldrich Chemicals Pvt Ltd., USA. Melting points were determined on an electrically heated VMP-III melting point apparatus. The FT-IR spectra were recorded using KBr discs and Nujol on FT-IR Jasco 4100 infrared spectrophotometer. 1H NMR spectra were recorded using Bruker DRX 400 spectrometer at 400 MHz with TMS as an internal standard. Mass spectra were recorded on LC-MS/MS (API-4000) mass spectrometer. Further, the elemental analysis of the compounds was performed on a Perkin Elmer 2400 elemental analyzer. The synthesis of proposed compound 2-methylxanthen-9-one was outlined in the scheme 1. The starting material phenyl benzoates (3) were prepared according to a reported procedure through the reaction of p-cresol (1) with benzoyl chloride (2) in the presence of 10% sodium hydroxide as a base. Compound 3 on subjected to Fries rearrangement afforded substituted diaryl methanone commonly known as hydroxy benzophenone (4). Compound 4 on reaction with ethyl chloroacetate resulted in ethyl [2-benzoyl-4-methylphenoxy] acetate (5) in excellent yield. Further, Compounds 5 with 10% NaOH in the presence of ethyl alcohol after refluxing gave 2-methylxanthen-9-one (6).

### Preparation of 4-methyl phenyl benzoate:

4-Methyl phenyl benzoate (3) was synthesized by benzoylation of 4-methyl phenol (1) with benzoyl chloride (2,1:1) using 10% sodium hydroxide solution. The reaction mass was stirred for about 2-3 hours at 0°C. The reaction was monitored by TLC using 4:1 n-hexane: ethyl acetate as a mobile phase. After completion of the reaction the oily product was extracted with ether layer. Ether layer was washed with 10% sodium hydroxide solution (3x50 ml) followed by water (3x30 ml) and then dried over anhydrous sodium sulphate and evaporated the solvent under pressure to afford compound 3 [14]. A pale-yellow liquid with 90% yield was obtained. IR (Neat): 1715 cm-1 (C=O). 1H NMR (DMSO): δ 2.45 (s, 3H, Ar-CH3), 7.5-8.2 (m, 9H, Ar-H). MS: m/z 213 (M+1). Anal. Calcd. for C14H12O2 (212): C, 79.22; H, 5.70. Found: C, 79.18; H, 5.76 %.

### Preparation of (2-Hydroxy-5-methylphenyl) phenyl methanone:

(2-Hydroxy-5-methylphenyl) phenyl methanone commonly known as hydroxy benzophenone (4) was synthesized by Fries rearrangement. Compound 3 (0.001 mol) was treated with anhydrous aluminum chloride (0.002 mol) as a catalyst at 150-170°C temperature without the presence of solvent for about 2-3 hours. The reaction mixture was then cooled to room temperature and quenched with 6N HCl in the presence of ice water. The reaction mixture was stirred for about 2-3 hours, filtered to separate solids and re-crystallized with methanol to obtain (2-hydroxy-5-methylphenyl) phenyl methanone (4). Yield 85%, mp 81-83 OC; IR (Nujol): 1670 (C=O), 3545-3649 cm-1 (OH); 1H NMR (CDCl3) d: 2.3 (s, 3H, CH3), 6.85-7.75 (m, 8H, Ar-H), 12.05 (br s, 1H, OH); MS: m/z 212 (M+1). Anal. Calcd. for C14H12O2 (212): C, 79.24; H, 5.66. Found: C, 79.26; H, 5.64%.

### Procedure for the Preparation of ethyl [2-benzoyl-4-methylphenoxy] acetate:

Compound 5 was obtained by refluxing a mixture of compound 4 (0.013 mol) and ethyl chloroacetate (0.026 mol) in dry acetone (50 ml) and anhydrous potassium carbonate (0.019 mol) for 7-8 hours. The reaction mixture was cooled and solvent was removed by distillation. The residual mass was triturated with cold water to remove potassium carbonate and extracted with ether (3x50 ml). The ether layer was washed with 10% sodium hydroxide solution (3x50 ml) followed by water (3x30 ml) and then dried over anhydrous sodium sulphate and evaporated to dryness to obtain crude solid, which on recrystallization with ethanol afforded ethyl [2-benzoyl-4-methylphenoxy] acetate (5) [15]. Yield, 79%; mp 61-63 OC; IR (Nujol): 1664 (C=O), 1760 cm-1 (ester C=O); 1H NMR (CDCl3): d 1.2 (t, J = 7 Hz, 3H, CH3 of ester), 2.3 (s, 3H, CH3), 4.1 (q, J = 6 Hz, 2H, CH2 of ester), 4.5 (s, 2H, OCH2), 7.1-7.7 (m, 8H, Ar-H); MS: m/z 298 (M+1). Anal. Calcd. For C18H18O4 (298): C, 72.48; H, 6.04. Found: C, 72.46; H, 6.02%.

### Preparation of 2-methylxanthen-9-one:

2-Methylxanthen-9-one (6) was obtained by the reaction of ethyl [2-benzoyl-4-methylphenoxy] acetate (5), in the presence of NaOH as a base and ethyl alcohol as a solvent (50ml) refluxed for about 7-8 hours and then cooled. The reaction mixture was neutralized, solvent removed by distillation. The residual mass was washed with water and recrystallized by methanol, gave 2-methylxanthen-9-one (6). Yield 70%, m.p. 369-373 K; IR (Nujol):1665 cm-1 (C=O);1H NMR (CDCl3): δ 2.3 (s, 3H, Ar-CH3), 6.9-7.6 (bm, 7H, Ar-H); MS: m/z 211 (M+1). Anal. Cal. for C14H10O2 (210): C, 79.98; H, 4.79; Found: C, 79.94; H, 4.76%.

### Single X-ray crystallography data collection, solution, refinement, and structure elucidation:

**Figure 1:**
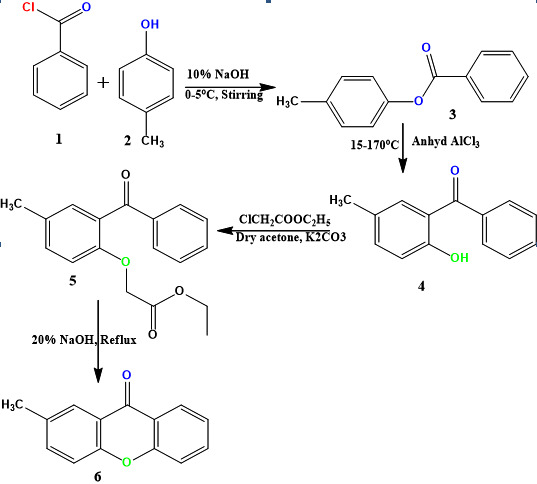
Synthesis of 2-methyl xanthen-9-one.

In compound 6 (Figure 1), all bond lengths were within normal ranges and comparable to those observed in the related structures. The three-ring system was not planar. The dihedral angle between the two benzene rings was 4.7 (1)°. pi-pi Interactions with distances Cg1...Cg2i = 3.605 (1) Å(symmetry code:1 - x, -y, -z); Cg2...Cg2i = 3.850 (1) Å and Cg3...Cg1ii = 3.580 (1) A [symmetry codes: (i) 1 - x, -y, -z; (ii) 2 - x, -y, 1 - z], Cg1, Cg2 and Cg3 are the centroids of C9/C14/C11-C13, C1-C4/C11/C14 and C5-C8/C13/C12 rings, respectively, from stacks of the molecules propagated. All H atoms were positioned geometrically and were treated as riding on their parent C atoms, with C-H distances of 0.93-0.96 Å; and with Uiso (H) = 1.2-1.5 Ueq (C).

### Data collection:

Oxford Diffraction Xcalibur Sapphire3 diffractometer, Radiation source: fine-focus sealed tube Graphite monochromator ω scans, Absorption correction: multi-scan (CrysAlis PRO; Oxford Diffraction, 2010) Tmin = 0.890, Tmax = 1.000, 10601 measured reflections, 2028 independent reflections, 1262 reflections with I > 2σ(I), Rint = 0.033, θmax = 26.0°, θmin = 3.6°, h = -10→10,k = -10→10, l = -10→10.

### Crystal data:

C14H10O2, Mr = 210.22, Triclinic, P1, Hall symbol: -P1, a = 8.2678 (7) A, b = 8.5268 (6) A, c = 8.5965 (7) A, α = 92.650 (6)°, β = 116.592 (8)°, γ = 104.045 (7)°, V = 517.28 (7) A3, Z = 2, F(000) = 220, Dx = 1.350 Mg m-3, Mo Kα radiation, λ = 0.71073 Å, Cell parameters from 3639, reflections, θ = 3.6-29.1°, μ = 0.09 mm-1, T = 293 K, Block, white, 0.30 x 0.20 x 0.20 mm.

### Refinement:

Refinement on F2, Least-squares matrix: full, R [F2 > 2σ (F2)] = 0.052, wR(F2) = 0.152, S = 1.04, 2028 reflections, 146 parameters, 0 restraints, Primary atom site location: structure-invariant, direct methods Secondary atom site location: difference Fourier Map, Hydrogen site location: inferred from neighboring sites, H-atom parameters constrained, w = 1/[σ 2(Fo2) + (0.0652P)2 + 0.0822P], where P = (Fo2 + 2Fc2)/3, (Δ/σ)max < 0.001, Δρmax = 0.13 e A-3, Δρmin = -0.15 e A-3.

The optimized structure of the title molecule is depicted in Figure 1b showing good agreement with the experimental results. This indicates the accuracy of B3LYP functional to predict the geometries of the organic compounds.

### In vitro antimicrobial activity:

The microbial strains were procured from the National Chemical Laboratory (NCL), Pune, India, and sub-cultured in our laboratory at optimum condition. The synthesized compound 6 (50μg/ml) was screened against the following Gram-positive (Streptococcus pyogenes, Staphylococcus aureus, Bacillus subtilis), Gram-negative (Salmonella typhimurium, Klebsiella pneumonia, Escherichia coli) and fungal strains (Candida albicans, Aspergillus niger and Aspergillus flavus). The zone of inhibition is determined by disc diffusion method [16] and MIC values were determined by broth dilution method, using ampicillin in case of bacterial, ketoconazole for fungal as standard control and the results are presented in Table 1 and Table 2.

### Docking studies:

The molecular interaction between protein and ligand can virtually be studied by subjecting the input files for molecular docking study. The docking operations can be performed by several algorithms, of which SURFLEX DOCK being such a program as available with SYBYL-X 2.1.1 software package (Tripos Inc. USA). The algorithm can readily be applied to rigid to flexible type docking. The input files as PDB file of protein and virtually sketched files of synthesized compounds were either collected from online server or drawn by using Chemdraw 15.0., and all other necessary calculations were performed as per default protocol [17]. Preamble to docking protocol, all the required input files were prepared, hydrogen atoms were added, water was removed, ionization state of C-terminal and N-terminal were fixed and finally energy minimized by steepest descent method applying Gasteiger-Marsili charges and MMFF94s forcefield to the protein file with 100 iterations of conjugate gradient method with 1.0 kcal/mol as the convergence criteria fixing to 0.5 dielectric constant, whereas, ligand files were converted to 3D forms and energy minimized by applying Gasteiger-Huckel charges. The Geom mode of Surflex-dock allows the ligand files to flexibly interact with rigid protein files. The binding site of the protein file was abstracted from co-crystal ligand bound information and protomol generation program uses such information to find suitable cavity for docking. The adopted docking protocol was validated using a comparison of the binding modes of co-crystalized ligand of the target protein FtsZ (PDB: 3VOB) before and after the docking study. Highlighting feature of Geom mode is generation of 20 conformers for each ligand, which interacts to the protein individually in order to identify the most stable conformer having best binding pose and binding energy. The respective ligand poses and docking scores in terms of Total score, Crash score and Polar score were obtained and represented accordingly.

### Density functional theory calculations (DFT):

The whole DFT calculations [18] were performed with B3LYP functional [19,20] under 6-31G(d,p) basis set by using Gaussian 09 [21] and Gauss View 0.5 programs [22]. DFT studies covered geometry optimization, Frontier molecular orbitals (FMOs): HOMO-LUMO energies and molecular electrostatic potential (MEP).

### Hirshfeld surfaces analysis and fingerprint plots:

The intermolecular interactions of the title molecule are deeply investigated by Hirshfeld surface analysis approach by using Crystal Explorer 17.5 program [23]. The normalization of contact distance (dnorm) (see eqn. (1)) permits us to investigate the intermolecular interactions regions, where di and de are the distance from the Hirshfeld surface to the nearest atom inside and outside the surface, respectively. The combination of de and di in the form of 2D fingerprint plot enables us to summaries the intermolecular contacts in the crystal lattice.

dnorm = (di -rivdw/ rivdw) + (de -revdw/revdw) → (1)

where rivdW and revdW being the van der Waals radii of the atoms.

## Results and Discussion:

**Figure 2:**
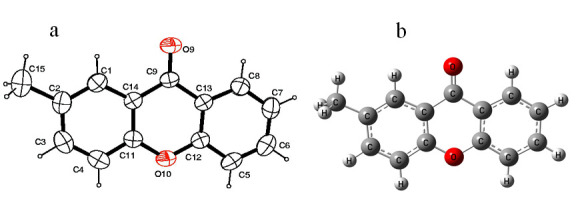
Structure of the title molecule: a) ORTEP with the atom-labelling scheme. The displacement ellipsoids are drawn at the 40% probability level. H atoms are shown as small spheres of arbitrary radii, and b) optimized structure at DFT/B3LYP/6-31G(d,p) level.

The structure of the newly synthesized compounds was established on the basis of 1H NMR, IR, mass, elemental analysis and X-ray diffraction studies. In IR spectra of benzoylated product (3), the emergence of O-C stretching band for ester group and in proton NMR the disappearance of broad singlet for OH proton of p-cresol (1) indicates the formation of compound (3). Further, fries' rearrangement of compound (3) gave 2-hydroxy benzophenone (4), which was confirmed by the disappearance of O-C stretching band and appearance of O-H stretching band in IR spectra. In addition, the appearance of broad singlet for OH proton and decrease in one aromatic proton in proton NMR also confirmed the product formation. Compound 4 on etherification with chloro ethylacetate gave substituted ethyl ester (5), which is confirmed by the disappearance of O-H stretching band and appearance of O-C stretching band for ester group in IR absorption spectra. Confirmation was also by observing the disappearance of broad singlet for OH proton and appearance of triplet and quartet for CH3, and CH2 protons respectively in proton NMR. Finally, the synthesis of compound 6 was confirmed by the appearance of C-O-C stretching in IR spectra and disappearance of singlet, triplet and quartet for CH2 and CH2 CH3 protons and decrease in one aromatic proton in 1HNMR spectrum (Figure 2) is highly evidenced. Further, in mass spectrum the m+ peak at 211.5 was exhibited in positive mode (Figure 2). In addition, the compound 6 was confirmed by X-ray crystal diffraction studies (Figure 1).

After confirmation of the newly prepared compound 6, they were tested against a panel of Gram-positive and Gram-negative bacteria as well as a few strains of fungi (Table 1 and Table 2) using ampicillin and ketoconazole as standards for bacteria and fungi, respectively. Compound 6 showed excellent antimicrobial activity against all test microorganisms. These positive results may be due to the presence of a fused three ring system with -CH3 and C=O groups in the new molecule and are in agreement with several previous studies [24] Because of the carbonyl group in compound 6 similar to that present in ampicillin and ketoconazole molecule, the effect could be comparable with that of the standard drug.

**Figure 3:**
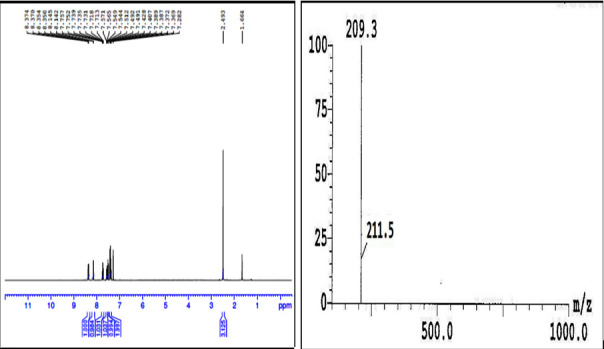
1HNMR and Mass Spectrum of compound 6.

A molecular docking study fundamentally defines the binding modes of ligand interaction at the active site of the receptor [17]. In our study the active Xanthone compound with notable antibacterial activity was subjected for docking studies against FtsZ protein and the crash score (revealing the inappropriate penetration into the binding site), Polar score (reports the polar region of the ligands) and total score was found to be -1.0241, 0.2314 and 5.6787 respectively. The inhibition of FtsZ (filamentous temperature sensitive protein Z) protein prevents the formation of divisome and hence it is a striking target for antibiotic research [25]. The binding interactions with the compounds revealed that compound finds well in the active pocket of the protein. The carbonyl oxygen of the chromone involves in p-donor hydrogen bonding with GLY196. The fused phenyl ring forms p-amide interaction with ASP199. The other phenyl ring involves in p-donor hydrogen and p-alkyl interaction with THR309 and LEU200 amino acid respectively. These interactions make the molecule to bind with protein molecule and hence responsible for showing desired activity (Figure 3).

**Figure 4:**
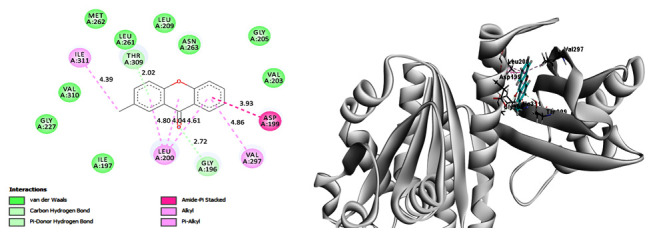
Binding pose of Xanthone with FtsZ protein (2D) and (3D) after docking studies.

The optimized structure of the title molecule using density functional theory with B3LYP functional under 6-31G (d,p) basis set is illustrated in Figure 1b. The comparison of optimized structure with the experimental results showed good agreement. This indicates the accuracy of B3LYP functional to predict the geometries of the organic compounds. The optimized structure has been taken as an input to predict the others DFT calculations as described below: The stability of the compounds is mainly influenced by FMOs (i.e., HOMO and LUMO), which donates the highest occupied and lowest unoccupied molecular orbitals, respectively [26]. The energy difference between HOMO and LUMO is referred as energy gap (δEgap). It plays a critical role in determining the molecular electrical transport properties and enables us to determine the kinetic stability, chemical reactivity, optical polarizability and chemical hardness-softness of a molecule [27]. Mostly small δEgap lead to the ease of transporting electrons from HOMO level to LUMO level. The surfaces of HOMO (ground state) and LUMO (1st excited state) are sketched in Figure 4. The HOMO orbitals exhibited the predominant of π character of electron density on the entire skeleton of molecule exception of the oxygen atoms that showed pure α character of electron density distribution. The highly concentration of π electron density over the rings resulted in multiple stacking interactions of title compound with different amino acids as shown in Figure 3, indicating the agreement between DFT and docking studies. On the other side, the electron density distribution over LUMO orbital showed mix of α and π character, but methyl group did not exhibit any type of electron density distribution. The HOMO and LUMO energies are found to be -6.275 and -1.880 eV, respectively. Hence, the calculated energy gap is 4.395 eV. The lower energy gap explains the charge transfer interactions taking place within the molecule, which recently, used to prove the bioactivity from intramolecular charge transfer [28]. Table 3 lists the energies of HOMO, LUMO, ΔEgap as well as other global reactivity parameters.

**Figure 5:**
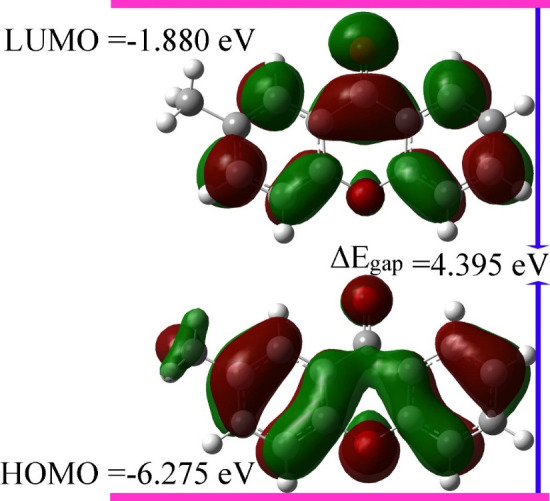
HOMO-LUMO orbitals with energy gap.

The mapping of MEP is a representation of the electrostatic potential mapped upon is surface of electron density, that illustrates the 3-D charge distributions of a molecule [29]. This information about the charge distributions enables us to predict how the molecule can interact with others. Figure 5 represents MEP of the title molecule with colours scaled from -7.166 e-2 (deepest red) to 7.166 e-2 (deepest blue), whereas the intermediary colours indicating the intermediary electrostatic potentials. The maximum negative electrostatic potential (electronegativity) appears around the oxygen atom of the carbonyl oxygen (O9), which indicate the nucleophilic site reaction of the molecule. This result is agreed with docking studies that showed formation of hydrogen bond between O9 and GLY196 residue (see Figure 3, Molecular docking studies). On the other hand, the maximum positive electrostatic potential appears around the hydrogen atoms of the phenyl ring especially H3 and H7 atoms, which indicate the electrophilic site reaction of the title molecule.

**Figure 6:**
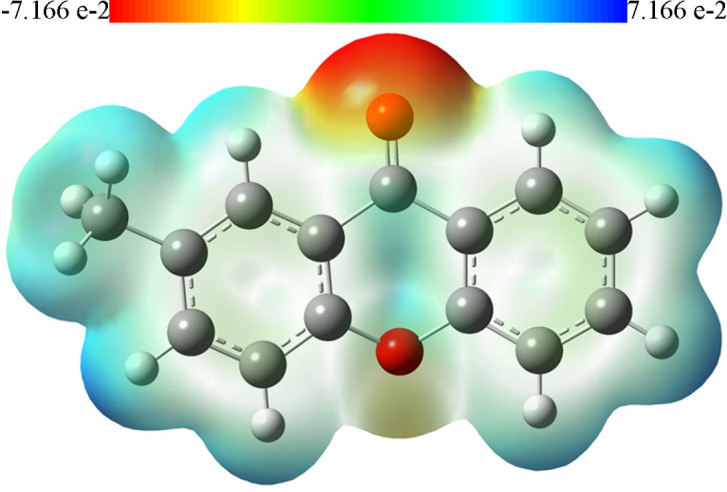
Molecular Electrostatic Potential (MEP) of the title compound.

**Figure 7:**
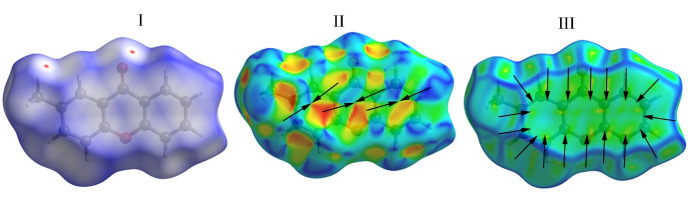
Hirshfeld surface with (I) dnorm, (II) Shape index and (III) Curvedness of the title molecule.

The Hirshfeld surface of the title molecule was mapped over volume of 253.15 Å3 and area of 246.12 Å2, respectively. Figure 6 present Hirshfeld surface over dnorm with colour range from -0.0185 a.u. (red) to 1.2012 a.u. (blue) represent the distance shorter and greater the sum of vdw radii of the contacted atoms, respectively. While the white color represents the distance equal to the sum of vdw radii. The dark red spots on the surface donate the hydrogen bonds. The fingerprint plots of the title molecule for all atoms are depicted in Figure 7a. Furthermore, the decomposition of fingerprint plots of a particular pair of close contact atoms are depicted in Figure 7b-d. H...H inter contact (Figure 7b) showed as a huge wing pointing at de = di ≈ 1.18 Å has an influence to the crystal packing with 49.1% contribution to the total Hirshfeld surface. While the H...O/O...H intermolecular interaction (Figure 7) comprising only 19.2% of Hirshfeld surfaces and appear as two characteristic wings have the major contribution toward crystal packing where the atoms contacted at distance less than the sum of vdw radii (i.e., de + di ≈ 2.66 Å). H..C/...H intermolecular interaction coated 15.5% of the Hirshfeld surface without significant contribution to the crystal packing of the title molecule. The contribution from C…C contacts (13.8%) (Figure 7) confirms the presence of π...π stacking in the crystal packing formation. This also could be shown on the shape index surface as red and blue triangles (Figure 6II) and also, as flat regions upon the curvedness surface (Figure 6 III).

## Conclusion

We document the design, synthesis, docking, Hirshfeld surface analysis and DFT calculations of 2-methylxanthen-9-with the FtsZ protein (PDB ID: 3VOB) from Staphylococcus aureus for antimicrobial applications.

## Figures and Tables

**Table 1:** Zone of inhibition of compound 6 against different bacteria and fungi at 1000 g/mL concentration.

	Zone of inhibition (in mm)
Compounds	Gram-positive bacteria	Gram-negative bacteria	Fungi
	Streptococcus pyogenes	Staphylococcus aureus	Bacillus subtilis	Salmonella typhimurium	Klebsiella pneumonia	Escherichia coli	Candida albicans	Aspergillus niger	Aspergillus flavus
Compound 6	33	30	37	42	41	40	41	43	39
Ampicillin	33	33	38	43	42	40	-	-	-
Ketoconazole	-	-	-	-	-	-	41	44	41

**Table 2:** Minimum inhibitory concentration of compound 6 against different bacteria and fungi.

	Zone of inhibition (in mm)
Compounds	Gram-positive bacteria	Gram-negative bacteria	Fungi
	Streptococcus pyogenes	Staphylococcus aureus	Bacillus subtilis	Salmonella typhimurium	Klebsiella pneumonia	Escherichia coli	Candida albicans	Aspergillus niger	Aspergillus flavus
Compound 6	2.5	5	10	15	10	15	20	10	20
Ampicillin	25	10	20	10	10	15	-	-	-
Ketoconazole	-	-	-	-	-	-	25	15	15

**Table 3:** HOMO-LUMO and global chemical parameter values of the title molecule.

Property	Chemical parameters	Value
HOMO energy	EH (eV)	-6.275
LUMO energy	EL (eV)	-1.88
Energy gap	δEgap = EL - EH (eV)	4.395
hardness	η = (EL - EH)/2	2.198
Softness	σ = 1/η	0.455
Chemical potential	μ = (EL + EH)/2	4.078
Electronegativity	ϒ = - σ	-4.078
Electrophilicity	ω = μ^2^/2 σ	3.783

**Figure 8:**
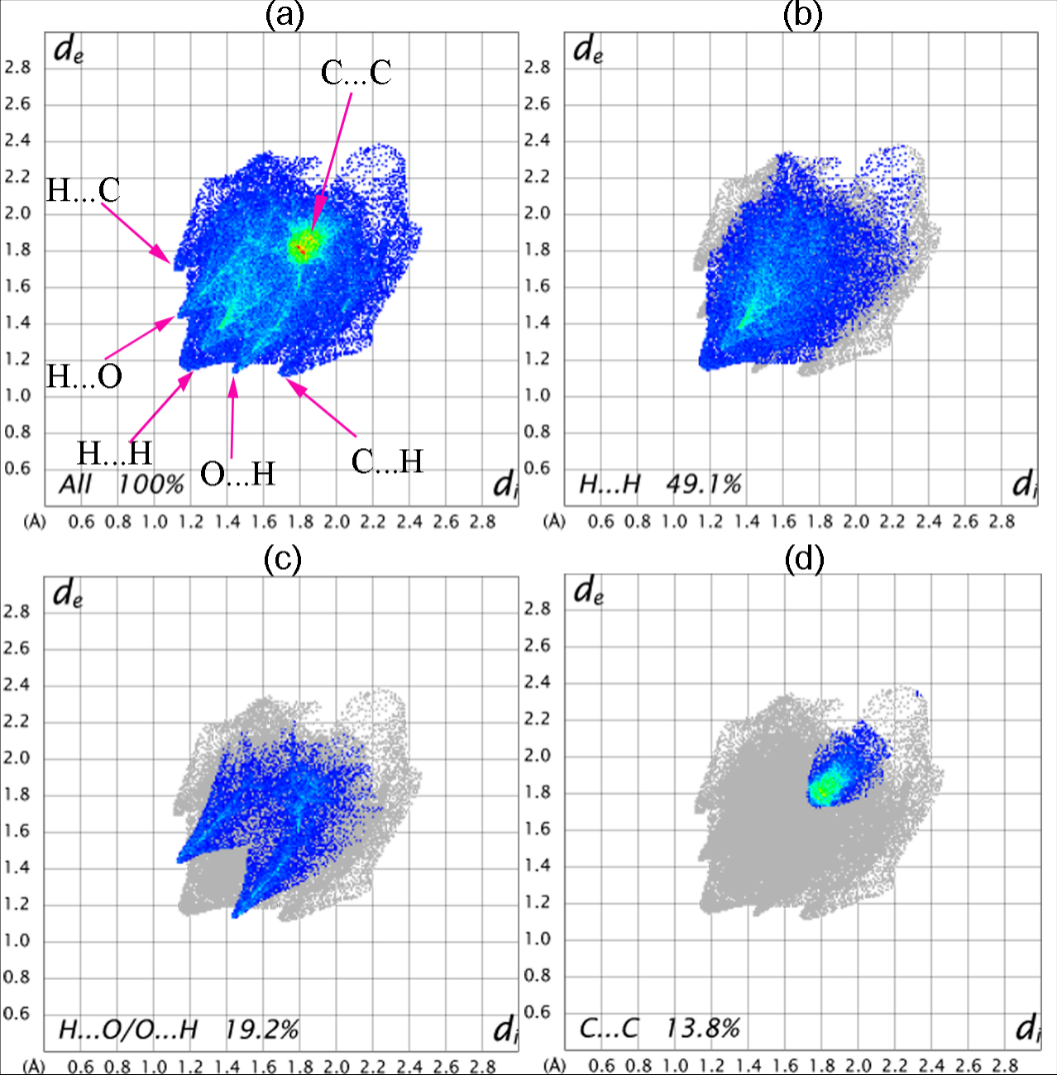
2D fingerprint of the title compound: a) Overall contacts and (b-d) represent decomposition of the contacts of particular pairs of atoms.
